# COVID-19, burnout, and healthcare-associated infections: a focus on wellness as a top safety priority

**DOI:** 10.1017/ash.2023.191

**Published:** 2023-09-11

**Authors:** Amie Patrick, Rachel Pryor

**Affiliations:** 1 Healthcare Infection Prevention Program, Virginia Commonwealth University Health Systems, 1250 East Marshall Street Suite 2-100 Richmond, VA 23298, USA; 2 Quality & Safety Department, Virginia Commonwealth University Health Systems, 1250 East Marshall Street Richmond, VA 23298, USA

## Introduction

The COVID-19 pandemic created physical and emotional burdens on healthcare workers (HCWs) and impacted them on both personal and professional levels. The increase in HCW responsibilities included the adoption of new safety and treatment protocols, higher patient acuities, and overcoming severe patient care staffing shortages. We reflect on healthcare worker burnout, patient safety, and infection prevention during the COVID-19 pandemic and propose the prioritization of healthcare worker wellness and resilience, on both individual and organizational levels, as an important driver of health system safety.

### Healthcare worker burnout: from bad to worse

Healthcare worker burnout is associated with symptoms of exhaustion, depression, insomnia, post-traumatic stress disorder (PTSD), or other mental health symptoms and is considered a national crisis and priority by the United States Surgeon General.^1^ About 50% of physicians reported symptoms of burnout prepandemic and burnout increased as the pandemic evolved.^1,2^ Burnout drives turnover in healthcare professions.^2,3^ The American Nurses Foundation reported that 31% of nurses under 35 expressed the intent to leave and 35 to 45% of all nurses are experiencing burnout.^3,4^ There are several factors that led to increased burnout during the pandemic. Healthcare workers were afraid of infecting family members and were challenged by ongoing changes in pandemic-driven patient care protocols.^5^ Healthcare workers faced an increase in workloads and inadequate supplies.^6^ Other reported drivers of burnout include decrease in social support, increased periods of isolation, and inadequate rest.^6^ Nurses reported moral distress from the poor prognosis of their patients. An increase in stress occurred from nurses who reported inadequate support from leaders and a lack of teamwork.^7^ There is a direct correlation between burnout and patient outcomes. This extreme level of stress and psychological pressure resulted in PTSD, anxiety, insomnia, and depression symptoms among healthcare workers. COVID-19 aggravated burnout that already existed within the healthcare systems prepandemic.^8^ Providers are not exempt. Physicians experiencing burnout can suffer substance abuse, depression, suicidal ideation, decreased self-care, and increased risk for motor vehicle crashes.^9^ All healthcare disciplines suffered the negative consequences of the pandemic.

Healthcare worker burnout impacts patient care and further demoralizes them. Patients experience longer recovery times when providers suffer from depersonalization.^9^ Medical errors increase stress on healthcare workers worsening burnout symptoms.^9^ Moral injury from errors deepens the existing symptoms of burnout.^4^ Thus, burnout causes a decrease in quality care, an increase in medical errors, increased recovery times for patients, and lower patient satisfaction scores. Burnout is associated with lower healthcare productivity.^9^ Healthcare workers are more effective when burnout is minimized.

### COVID-19, patient safety and healthcare infection prevention

Patient safety is the top priority in patient care as *first, do no harm* is the primary tenet of medicine. Healthcare workers suffering from burnout pose an increased risk of accidental harm to patients.^4,9^ Failure to minimize avoidable harm increases the risk of patient and family suffering caused by death, disability, increased cost, and longer length of stay.^10^ Healthcare organizations maximize patient safety through targeted processes of care measures, technologies, and desired behaviors to mitigate the risk of adverse events.^10^ Healthcare workers experiencing burnout may suffer from greater depersonalization, be less connected with a safety mission, and may be more prone to errors and breaks in safety protocols (Table 1).

**Table 1.** Key Factors for Healthcare Worker Wellness, Resilience, Patient Safety, and Infection Prevention

Theme	Comments
HCW burnout and patient safety	HCWs experiencing burnout may suffer from greater depersonalization, be less connected with a safety mission, and may be more prone to errors and breaks in safety protocols
HCW burnout and infection prevention	The COVID-19 pandemic and heightened HCW burnout are correlated with increases in CLABSIs, CAUTIs, VAEs, and MRSA infections
Individual level interventions	Deliberate activities such as mindfulness meditation, exercise, and reflection
System level interventions	Organizational strategies focused on engagement, social support, collegiality, adequacy of resources, optimizing workflows, and providing HCW mental health assistance
HCW wellness as the *primary factor* in patient safety	Infection prevention leaders should advocate for institutional wellness – much like advocating for infection prevention programs and resources. Wellness of the infection prevention team drives engagement and infection prevention practice, an important aspect of the global patient safety mission

**Figure 1.**
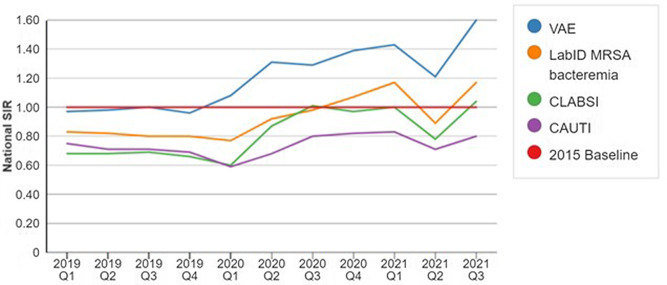
Quarterly National SIRs for select HAI types, 2019-Q1–2021-Q3^11^.

The Centers for Disease Control and Prevention reported a surge in healthcare-associated infection (HAI) during the COVID-19 pandemic (Figure 1).^11^ These include a higher incidence of ventilator-associated events (VAE), central line-associated bloodstream infections (CLABSI), catheter-associated urinary tract infections (CAUTI), and methicillin-resistant *Staphylococcus aureus* (MRSA) bacteremia when comparing pandemic years to the most recent year prior to the pandemic.

The proportionate impact of HCW burnout on HAI outcomes remains undefined, though Cimiotti et al reported that nurse burnout was significantly associated with an increased risk for urinary tract infections and surgical site infections.^12^ Various studies demonstrate that burnout is associated with heightened risks for patient safety incidents, though not specifically HAIs.^13–15^ We argue that the unique HCW burnout experienced during the COVID-19 pandemic, coupled with factors outlined below, created a “perfect storm” of conditions that made patients increasingly vulnerable to HAIs during this period.

Various forces potentially drove the HAI increase during the COVID-19 pandemic. Hospital-associated infections likely heightened secondary to changes in hospital procedures resulting in increasing intravenous (IV) tubing length.^16^ This was done so IV medications could be changed outside of patient rooms, and to allow nurses to bundle care to decrease overall personal protective equipment (PPE) usage. Staffing shortages played a role in increasing HAIs rates secondary to staff fatigue and burnout, resulting in greater reliance on third-party agency staffing.^17,18^ Agency nurses may be less knowledgeable of institution-specific safety culture awareness and hospital-specific HAI prevention techniques.^19,20^

### Individual and organizational wellness and the path toward resiliency

No single intervention exists to minimize burnout and heighten healthcare worker wellness. There are various individual and organizational strategies to promote resiliency and decrease burnout.^5^ Individuals can use self-guided coping mechanisms to reduce signs and symptoms of stress. This includes mindful activities, physical activity, meditation, and reflection.^21–23^ Organizational strategies are more impactful at reducing the overall signs and symptoms of burnout within systems.^23,24^ Organizational strategies include improving and reducing workloads, workflows, increasing flexibilities of schedules to improve work-life integration, provide opportunities for advancement, to bring meaning in work, promote collegiality within the organization to increase social support, and to decrease the stress of the organization as a whole.^2,23^ For example, a positive work environment for front-line providers includes supportive leadership, the ability to balance a work schedule, professional autonomy, adequate resources, and opportunities to professionally advance.^8^

Various professional and government organizations endorse wellness enhancement tools. The American Hospital Association published a Well-Being Playbook 2.0 to serve as an executive guide for hospital leaders.^25^ The Institute for Healthcare Improvement toolkit, titled, *Guide to Promoting Health Care Workforce Well-Being During and After the COVID-19 Pandemic* serves a similar purpose.^26^ The Office of the Assistant Secretary for Preparedness and Response that serves under the Health and Human Services published tips for resiliency strategies and workforce retention, *Creating a Caring Workforce Culture: Practical Approaches for Hospital Executives.* ^27^ The U.S. Surgeon General made HCW burnout top five priorities.^28^ Additional research is needed on the direct impact of hospital-acquired infections and burnout.

A multi-path approach is required to minimize HCW burnout, maximize resilience of the workforce, and maximize patient safety. First, healthcare systems must prioritize the recognition of burnout including depersonalization, emotional exhaustion, and personal lack of accomplishment, and prepare for future pandemics and global health crises.^6,29^ Healthcare workers must be aware that their organization is working to improve and provide a healthy professional environment to feel supported.^30^ Addressing burnout cannot be done on the individual level alone.^2,3^ Organizations must assess HCWs regularly to identify dimensions of burnout and well-being.^2,29^ Professional engagement or worker motivation is a healthy way to reduce burnout and increase safety within an organization.^29,31^ Healthcare organizations must also consistently provide mental health support to their workforce.^1^

## Conclusion

Healthcare worker burnout worsened during the COVID-19 pandemic, deteriorating from bad to worse. The COVID-19 pandemic also resulted in increased HAIs. Although the proportionate impact of healthcare worker burnout on HAI outcomes is not well defined, burnout likely increases depersonalization and decreases safety practices across an organization, including infection prevention protocols. This may result in decreased fidelity with standard, horizontal infection prevention protocols and heighten device-associated infection risk.

Patient safety outcomes are a general reflection of organizational safety culture and overall institutional performance, this includes healthcare worker engagement and wellness. First and foremost, in the safety mission, is the wellness of the healthcare workforce. Institutions with established wellness programs are committed to healthcare worker engagement, regularly measure wellness, and use targeted interventions to minimize burnout. Top institutions are also committed to the study of drivers and mitigators of healthcare worker burnout. Individual resilience is also valuable. Examples and guidance for maximizing HCW resilience exist in the published literature. Collectively, these individual and system-level interventions impact healthcare worker wellness greater than when instituted in isolation.

It is time to focus on HCW wellness as the *primary factor* in patient safety. Infection prevention leaders should be aware of institutional wellness initiatives and relentlessly advocate for their ongoing implementation and expansion. This is both for the wellness of the infection prevention team, which drives infection prevention practice, but also for heightened, global patient safety, which further impacts patient-centered outcomes. Advocating for healthcare worker wellness, much like advocating for improvements in hand hygiene and safety checklists, is critical for the desired safety outcomes and gets us closer to the shared goal of minimal preventable harm to patients.
